# Un nodule rose du visage

**DOI:** 10.11604/pamj.2017.27.205.13273

**Published:** 2017-07-18

**Authors:** Amina Kissou, Badredine Hassam

**Affiliations:** 1 Service de Dermatologie, Centre Hospitalier Universitaire Ibn Sina, Rabat, Maroc

**Keywords:** Nodule, visage, histiocytofibrome, Nodule, face, histiocytofibroma

## Abstract

We report the case of a 32-year old female patient, with no notable medical history, requiring dermatology consultation for evaluation of a nodule on her right cheek which had evolved over the past 10 years. Clinical examination showed a reddish dermal-based nodule with a smooth surface. The lesion measured 1cm in diameter and was located at the level of the right cheek (A). There was no adenopathy and the remainder of the clinical examination was normal. The patient underwent skin biopsy which showed tumor proliferation composed of fusiform cells with poorly limited eosinophilic cytoplasm and lightly atypical elongated nuclei without mitosis and with mononuclear inflammatory cell infiltrate at the level of the dermis. The epidermis was thinner. Anti-CD68 antibody was positive, while anti-CD34 antibody, PS100 and anti-AML were negative. The diagnosis of benign cellular histiocytofibroma was retained. The patient underwent total resection with a healthy resection margin of 5mm. The patient had a median 2-year follow-up with no recurrences identified. Benign histiocytofibroma mainly occurs in middle-aged women. It more often appears as an erythematous nodular, bluish, brownish or achromique dermal-based little painful but sometimes embarrassing lesion characterized by firm consistency and commonly located at the level of the lower limbs. Racial histiocytofibroma is rarely reported in the literature. Differential diagnosis includes Darier-Ferrand dermatofibrosarcoma, leiomyoma, Kaposi nodule and solitary fibrous tumor of the skin. Histologically, benign cellular histiocytofibroma is composed of pure intradermal disordered proliferation of fusiform cells arranged in bundles or in eddies and circumscribed by lymphocytic inflammatory reaction with presence of foamy histiocytes. The lesion is often highly vascularized with possibile hemorrhagic foci and especially, with angiogenesis images. In a minority of cases, especially in the case of huge histiocytofibromas, the epidermis is thinner and may even ulcerate. The immunohistochemistry shows the expression of CD68 and F XIIIa + positive cells while a lack of CD34, PS100 and Anti-AML expression. It is characterized by a chronic, benign evolution with possible spontaneous regression. The treatment is based on surgical resection.

## Image en médecine

Une jeune patiente âgée de 32 ans, sans antécédents pathologiques notables, s'est présentée en consultation de dermatologie pour un nodule de la joue droite qui évoluait depuis 10 ans. L'examen clinique trouvait un nodule rougeâtre à surface lisse avec une base enchâssée dans le derme. La lésion mesurait 1cm de diamètre et siégeait au niveau de la joue droite (A). Il n'y avait pas d'adénopathie et le reste de l'examen clinique était normal. Une biopsie cutanée a été réalisée et avait montré au niveau du derme, une prolifération tumorale faite de cellules fusiformes à cytoplasme éosinophile mal limité et aux noyaux allongés peu atypiques sans mitose avec un infiltrat inflammatoire mononuclées. L'épiderme était aminci. Les anti-corps anti CD68 était positif, par contre, les anti-corps anti CD34, PS100 et anti AML étaient négatifs. Le diagnostic d'histiocytofibrome bénin cellulaire a été retenu. La patiente a bénéficié d'une exérèse totale avec une marge de 5mm qui étaient saines. Il n y avait pas de récidive avec un recul de 2 ans. L'histiocytofibrome bénin est une tumeur qui se voit fréquemment chez la femme d'âge moyen. Elle se présente le plus souvent sous forme nodulaire érythémateuse, bleutée, brunâtre ou achromique, caractérisée par sa consistance ferme, enchâssée dans le derme, peu douloureuse mais parfois gênante avec une localisation typique au niveau des membres inférieurs. La localisation au niveau du visage comme notre patiente est rarement rapporté. Le diagnostic différentiel se pose avec un dermatofibrosarcome de Darier et Ferrand, un léiomyosarcome, un léiomyome, un nodule de Kaposi et avec la tumeur fibreuse solitaire dans sa localisation cutanée. Histologiquement, l'histiocytofibrome bénin dans sa forme cellulaire est formé d'une prolifération intradermique pure, désordonnée, faite de cellules fusiformes disposées en faisceaux ou en tourbillons et circonscrites par une réaction inflammatoire lymphocytaire avec présence d'histiocytes spumeux. La lésion est souvent richement vascularisée avec possibilité de foyers hémorragiques et surtout images d'angiogénèse. Dans une minorité de cas, concernant surtout les histiocytofibromes de grande taille, l'épiderme est aminci et peut même s'ulcérer. L'immunohistochimie montre un marquage positif du CD68 et F XIIIa+, par contre, le CD34, PS100 et Anti-AML sont négatifs. L'évolution est chronique, bénigne avec possibilité de régression spontanée. Le traitement repose sur l'exérèse chirurgicale.

**Figure 1:**
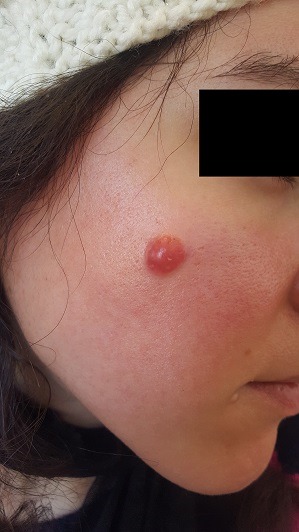
Un nodule rose du visage

